# Supplementary material for: Molecular mechanism to target the endosomal Mon1-Ccz1 GEF complex to the pre-autophagosomal structure
Source: eLife. 2018 Feb 15;7:e31145. doi: 10.7554/eLife.31145 (PMC5841931; doi:10.7554/eLife.31145)
Supplement: Supplementary file 1 [file elife-31145-supp1.docx]

**Supplemental File 1a**

| **Strains** | **Genotype** | **Reference** |
| --- | --- | --- |
| BY4741 | MATa *his3∆1 leu2∆0 met15∆0 ura3∆0* | Euroscarf Library |
| BY4742 | MATalpha *his3Δ1 leu2Δ0 lys2Δ0 ura3Δ0* | Euroscarf Library |
| BY4732 | MATa  *his3∆200 leu2∆0 met15∆0 trp1∆63 ura3∆0* | Euroscarf Library |
| SEY6210 | MATalpha *leu2-3 leu2-112 ura3-52 his3-∆200 trp1-∆101 lys2-801 suc2-∆9 GAL* | Reggiori F |
| CUY2470 | BY4732; *CCZ1::TRP1-GAL1pr MON1::HIS3MX6-GAL1pr CCZ1::TAP-URA3* | Nordmann  et al., 2010 |
| CUY4096 | BY4732; *CCZ1::TRP1-GAL1pr MON1::HIS3MX6-GAL1pr CCZ1(1-168aa)::TAP-URA3* | Nordmann  et al., 2010 |
| CUY9508 | BY4732; *MON1::HIS3MX6-GAL1pr CCZ1::hphNT1 pRS406-GAL1pr-CCZ1::URA TAP::KanMx* | This study |
| CUY10059 | BY4741; *vam3∆::kanMX*  *MON1::URA3-PHO5pr-GFP-myc mCherry-ATG8:: natNT2* | This study |
| CUY10062 | SEY6210; *vps11-1::HA-HIS3 CCZ1::URA3-PHO5pr-GFP-myc mCherry-ATG8:: natNT2* | This study |
| CUY10063 | SEY6210; *vps11-1::HA-HIS3 MON1::URA3-PHO5pr-GFP-myc mCherry-ATG8:: natNT2* | This study |
| CUY10147 | BY4732; *MON1::HIS3MX6-GAL1pr CCZ1::hphNT1*  *pRS406-GAL1pr-CCZ1(Y236A V239A Y445A L448A)::URA TAP::KanMX* | This study |
| CUY10157 | BY4741; *vam3∆::kanMX CCZ1::URA3-PHO5pr-GFP-myc mCherry-ATG8:: natNT2 atg1∆ :: hphNT1* | This study |
| CUY10158 | BY4741; *vam3∆::kanMX CCZ1::URA3-PHO5pr-GFP-myc mCherry-ATG8:: natNT2 atg9∆ :: hphNT1* | This study |
| CUY10161 | BY4741; *vam3∆::kanMX CCZ1::URA3-PHO5pr-GFP-myc mCherry-APE1:: natNT2* | This study |
| CUY10162 | BY4741; *vam3∆::kanMX CCZ1::URA3-PHO5pr-GFP-myc mCherry-APE1:: natNT2 atg1∆ :: hphNT1* | This study |
| CUY10164 | BY4741; *vam3∆::kanMX CCZ1::URA3-PHO5pr-GFP-myc mCherry-APE1:: natNT2 atg8∆ :: hphNT1* | This study |
| CUY10168 | BY4741 *vam3∆::kanMX CCZ1::URA3-PHO5pr-GFP-myc atg4∆ :: hphNT1 mCherry-APE1:: natNT2* | This study |
| CUY10169 | BY4741; *vam3∆::kanMX CCZ1::URA3-PHO5pr-GFP-myc atg12∆ :: hphNT1 mCherry-APE1:: natNT2* | This study |
| CUY10170 | BY4741; *vam3∆::kanMX CCZ1::URA3-PHO5pr-GFP-myc atg16∆ :: hphNT1 mCherry-APE1:: natNT2* | This study |
| CUY10191 | BY4741; *YPT7::HIS5-PHO5pr-Myc-GFP mCherry-ATG8:: natNT2* | This study |
| CUY10192 | BY4741; *vam3∆::kanMX YPT7::URA3-PHO5pr-GFP-myc mCherry-ATG8:: natNT2* | This study |
| CUY10193 | BY4741; *vam3∆::kanMX vam3ts::MET15 mCherry-ATG8:: natNT2* | This study |
| CUY10194 | BY4741; *CCZ1::URA3-PHO5pr-GFP-myc mCherry-APE1:: natNT2* | This study |
| CUY10195 | BY4741; *YPT7::HIS5-PHO5pr-Myc-GFP mCherry-APE1:: natNT2* | This study |
| CUY10196 | BY4741; *vam3∆::kanMX Ypt7::URA3-PHO5pr-GFP-myc mCherry-APE1:: natNT2* | This study |
| CUY10197 | BY4741; *vam3∆::kanMX YPT7::URA3-PHO5pr-GFP-myc mCherry-APE1:: natNT2 atg1∆ :: hphNT1* | This study |
| CUY10198 | BY4741; *vam3∆::kanMX YPT7::URA3-PHO5pr-GFP-myc mCherry-APE1:: natNT2 atg4∆ :: hphNT1* | This study |
| CUY10199 | BY4741; *vam3∆::kanMX YPT7::URA3-PHO5pr-GFP-myc mCherry-Ape1:: natNT2 atg8∆ :: hphNT1* | This study |
| CUY10200 | BY4741; *vam3∆::kanMX YPT7::URA3-PHO5pr-GFP-myc mCherry-APE1:: natNT2 atg16∆ :: hphNT1* | This study |
| CUY10470 | SEY6210; *mCherry-ATG8::natNT2 mon1∆::HIS pRS406-NOP1pr-GFP-MON1(1-319)::URA* | This study |
| CUY10471 | SEY6210; *mCherry-ATG8::natNT2 mon1∆::HIS pRS406-NOP1pr-GFP-MON1::URA* | This study |
| CUY10472 | SEY6210; *mCherry-ATG8::natNT2 mon1∆::HIS pRS406-NOP1pr-GFP-MON1(158-end)::URA* | This study |
| CUY10478 | SEY6210; *mCherry-ATG8::natNT2 ccz1∆::hphNT1 pRS406-NOP1pr-GFP-CCZ1::URA* | This study |
| CUY10479 | SEY6210; *mCherry-ATG8::natNT2 ccz1∆::hphNT1 pRS406-NOP1pr-GFP-CCZ1(1-162)::URA* | This study |
| CUY10484 | SEY6210; *mCherry-ATG8::natNT2 ccz1∆::hphNT1 pRS406-NOP1pr-GFP-CCZ1(Y236A L239A)::URA* | This study |
| CUY10485 | SEY6210; *mCherry-ATG8::natNT2 ccz1∆::hphNT1 pRS406-NOP1pr-GFP-CCZ1(Y445A L448A)::URA* | This study |
| CUY10486 | SEY6210; *mCherry-ATG8::natNT2 ccz1∆::hphNT1 pRS406-NOP1pr-GFP-CCZ1(Y236A V239A Y445A L448A)::URA* | This study |
| CUY10672 | BY4732; *MON1::HIS3MX6-GAL1pr ccz1∆::hphNT1 GAL::GAL-CCZ1(Y236A V239A) TAP::KANMX* | This study |
| CUY10675 | SEY6210; *mCherry-ATG8::natNT2 ccz1∆::hphNT1 MUP1-GFP::HIS pRS406-NOP1pr-CCZ1::URA* | This study |
| CUY10676 | SEY6210; *mCherry-ATG8::natNT2 ccz1∆::hphNT1 MUP1-GFP::HIS pRS406-NOP1pr-CCZ1(Y236A V239A)::URA* | This study |
| CUY10677 | SEY6210; *mCherry-ATG8::natNT2 ccz1∆::hphNT1 MUP1-GFP::HIS pRS406-NOP1pr-CCZ1(Y445A L448A)::URA* | This study |
| CUY11056 | SEY6210; *vps21∆::hphNT1 CCZ1:: HIS-PHO5pr-GFP-myc* | This study |
| CUY11058 | SEY6210; *VPS21::KanMX-ADHpr VPS8::natNT2-TEFpr mCherry-ATG8::hphNT1* CCZ1:: *URA3-PHO5pr-GFP-myc* | This study |
| CUY11059 | BY4741; *mCherry-APE1:: natNT2* | This study |
| CUY11061 | SEY6210; *atg8∆::natNT2* | This study |
| CUY11063 | BY4741; *atg14∆::kanMX mCherry-APE1::hph* | This study |
| CUY11065 | BY4741; *CCZ1::TAP-URA3 GFP-ATG8::natNT2* | This study |
| CUY11069 | SEY6210; *VPS11-1::HA-HIS3 MUP1-GFP::TRP* | This study |
| CUY11072 | BY4741; *CCZ1::URA3-PHO5pr-GFP-myc mCherry-ATG8:: natNT2 vps21∆::hphNT1* | This study |
| CUY11073 | BY4741; *vam3∆::kanMX CCZ1::URA3-PHO5pr-GFP-myc mCherry-APE1:: natNT2 atg2∆::hphNT1* | This study |
| CUY10174 | BY4741; *vam3∆::kanMX CCZ1::URA3-PHO5pr-GFP-myc mCherry-APE1:: natNT2 atg9∆::hphNT1* | This study |
| CUY10175 | BY4741; *vam3∆::kanMX CCZ1::URA3-PHO5pr-GFP-myc mCherry-APE1:: natNT2 atg18∆::hphNT1* | This study |
| CUY11079 | BY4741; *vam3∆::kanMX mCherry-APE1::natNT2 atg14∆::hphNT1 LEU2::pCu-GFP-ATG8* | This study |
| CUY10489 | BY4742; *pho13∆::kanMX pho8::PHO8∆60* | Reggiori F |
| CUY10490 | BY4742; *pho13∆::kanMX pho8::PHO8∆60 atg9∆::URA* | Reggiori F |
| CUY11199 | BY4742; *pho13∆::kanMX pho8::PHO8∆60 atg8∆::natNT2* | This study |
| CUY11200 | BY4742; *pho13∆::kanMX pho8::PHO8∆60 vps21∆::hphNT1* | This study |
| CUY11202 | SEY6210; *vps21∆::hphNT1* *atg8∆::natNT2* | This study |
| CUY11203 | BY4741; *mCherry-APE1:: natNT2 atg8∆::natNT2* | This study |
| CUY11204 | SEY6210; *atg8∆::natNT2 pRS406-NOP1pr-GFP-ATG8::NOP1pr* | This study |
| CUY11205 | SEY6210; *atg8∆::natNT2 pRS406-NOP1pr-GFP-ATG8 (I21R):: NOP1pr* | This study |
| CUY11206 | BY4741; *mCherry-APE1:: natNT2 atg8∆::natNT2 pRS406-NOP1pr-GFP-ATG8::NOP1pr* | This study |
| CUY11207 | BY4741; *mCherry-APE1:: natNT2 atg8∆::natNT2 pRS406-NOP1pr-GFP-ATG8 (I21R)::NOP1pr* | This study |
| CUY11208 | BY4742; *pho13∆::kanMX pho8::PHO8∆60 atg8∆::natNT2 pRS406-NOP1pr-GFP-ATG8::NOP1pr* | This study |
| CUY11209 | BY4742; *pho13∆::kanMX pho8::PHO8∆60 atg8∆::natNT2 pRS406-NOP1pr-GFP-ATG8 (I21R)::NOP1pr* | This study |
| CUY11212 | BY4741; *vam3∆::kanMX vam3ts::MET15 mCherry-ATG8:: natNT2 GFP-CCZ1::URA vps21∆::hphNT1* | This study |
| UY11213 | BY4741; *CCZ1::TAP-URA3 GFP-ATG8::natNT2 atg4∆::hphNT1* | This study |
