# Supplementary material for: Molecular mechanism to target the endosomal Mon1-Ccz1 GEF complex to the pre-autophagosomal structure
Source: eLife. 2018 Feb 15;7:e31145. doi: 10.7554/eLife.31145 (PMC5841931; doi:10.7554/eLife.31145)
Supplement: Supplementary file 2 [file elife-31145-supp2.docx]

**Supplemental File 1b**

| **Plasmids** | **Reference** |
| --- | --- |
| pGEX-2TK-*UBIQUITIN* | Gift of F. Sauer |
| pGEX-4T-1-*ATG8* G116A R117∆ | Gift of Ivan Dikic |
| pETDuet-1 *ATG8-HIS* | Gift of Sascha Marten |
| pGEX-4T-3-*ATG8∆N8* | This study |
| pGEX-4T-3-*ATG8∆N24* | This study |
| pRS406-*NOP1pr-GFP-CCZ1* | Nordmann et al., 2010 |
| pRS406-*NOP1pr-GFP-CCZ1(Y236A V239A)* | This study |
| pRS406-*NOP1pr-GFP-CCZ1(Y445A L448A)* | This study |
| pRS406-*NOP1pr-GFP-CCZ1(Y236A V239A Y445A L448A)* | This study |
| pRS406-*GAL1pr-CCZ1* | Cabrera et al., 2014 |
| pRS406-*GAL1pr-CCZ1(Y236A V239A)* | This study |
| pRS406-*GAL1pr-CCZ1(Y236A V239A Y445A L448A)* | This study |
| pGEX-4T-3-*ATG8 (I21R)* | This study |
| pRS415*-pCuGFP-ATG8* | Gift of Fulvio Reggiori |
| pRS315*-CUP1pr-BFP-APE1* | Gift of Fulvio Reggiori |
| pRS416-*ATG8pr-GFP-ATG8* | Gift of Fulvio Reggiori |
| pRS406-*NOP1pr-GFP-ATG8* | This study |
| pRS406-*NOP1pr-GFP-ATG8 (I21R)* | This study |
